# Hydrocarbon degraders establish at the costs of microbial richness, abundance and keystone taxa after crude oil contamination in permafrost environments

**DOI:** 10.1038/srep37473

**Published:** 2016-11-25

**Authors:** Sizhong Yang, Xi Wen, Yulan Shi, Susanne Liebner, Huijun Jin, Amedea Perfumo

**Affiliations:** 1 State Key Laboratory of Frozen Soils Engineering (SKLFSE), Northwest Institute of Eco-Environment and Resources, Chinese Academy of Sciences (CAS), Lanzhou, 730000, China; 2 GFZ German Research Centre for Geosciences, Helmholtz Centre Potsdam, Section 5.3 Geomicrobiology, Telegrafenberg, 14473 Potsdam, Germany; 3 College of Electrical Engineering, Northwest University for Nationalities, Lanzhou, 730030, China

## Abstract

Oil spills from pipeline ruptures are a major source of terrestrial petroleum pollution in cold regions. However, our knowledge of the bacterial response to crude oil contamination in cold regions remains to be further expanded, especially in terms of community shifts and potential development of hydrocarbon degraders. In this study we investigated changes of microbial diversity, population size and keystone taxa in permafrost soils at four different sites along the China-Russia crude oil pipeline prior to and after perturbation with crude oil. We found that crude oil caused a decrease of cell numbers together with a reduction of the species richness and shifts in the dominant phylotypes, while bacterial community diversity was highly site-specific after exposure to crude oil, reflecting different environmental conditions. Keystone taxa that strongly co-occurred were found to form networks based on trophic interactions, that is co-metabolism regarding degradation of hydrocarbons (in contaminated samples) or syntrophic carbon cycling (in uncontaminated samples). With this study we demonstrate that after severe crude oil contamination a rapid establishment of endemic hydrocarbon degrading communities takes place under favorable temperature conditions. Therefore, both endemism and trophic correlations of bacterial degraders need to be considered in order to develop effective cleanup strategies.

Large reservoirs of crude oils are localised in permafrost-affected areas of the Arctic, particularly in Siberia and Alaska. For the last 50 years oil has been transported from these regions via large-diameter and long-distance pipelines such as the China-Russia crude oil pipeline (CRCOP), which crosses 441 km of discontinuous permafrost and 465 km of deep (>1.5 m) seasonally frozen ground^1^,^2^. Under such extreme environmental conditions, the risk of oil spillage is high due to occasional pipeline failures that can be caused by frost-heaving, thaw-settlement of pipeline foundations and erosion^2^. Oil spills from pipeline ruptures are the largest source of terrestrial petroleum pollution in the Arctic, and in the past some pipeline accidents have caused severe damage to cold ecosystems in northern Russia, Canada and Alaska^3^.

Several studies have investigated the bioremediation of contaminated cold soils both in the Arctic^4^,^5^,^6^ and Antarctica^7^,^8^,^9^. Although environmental conditions are challenging (e.g. low temperature, poor availability of liquid water, oligotrophic environment), cold soils and sediments are inhabited by a variety of microorganisms capable of degrading hydrocarbons both alkanes, aromatics and polycyclic aromatics. For example, bacteria belonging to the genera *Pseudomonas*, *Sphingomonas*, *Rhodococcus* and *Arthrobacter* have been frequently isolated from contaminated cold terrestrial ecosystems and are considered key-players in biodegradation processes^7^,^10^,^11^. More recently, the growing application of molecular and meta-omics technologies (e.g. meta-genomics, transcriptomics and proteomics) to biodegradation studies has been providing a more comprehensive view of microbial community structure, dynamics and functioning at the contaminated sites^12^,^13^. A few studies have used high-throughput DNA sequencing technologies to investigate the biodiversity of hydrocarbon degrading microorganisms in cold ecosystems, including Arctic soils contaminated with diesel^4^,^5^ or jet fuel^14^, and crude oil-contaminated permafrost^15^. These approaches have proved ideal to highlight at best community biodiversity structure and shifts in response to disturbance events such as oil spills. However, to advance further from the basic inventory description of the composition and diversity of microbial communities, new analytical tools that enable to infer also on biogeographical patterns, potential interactions between taxa, shared ecological niches, keystone microbial groups, network and non-random co-occurrence patterns amongst taxa should be prompted^16^. Network-based data analysis has recently been applied to large scale environmental sequencing in a few studies to gain insights, for examples, into microbial interactions in soil^17^,^18^ including upon contamination with crude oil^19^,^20^.

In particular research on permafrost soil bioremediation is at present under-represented, with only a few studies available on the topic^6^,^15^,^21^. In previous works^15^,^22^ we used a 16 S rRNA gene pyrosequencing approach to describe the bacterial biodiversity of uncontaminated/contaminated deep active layers and upper permafrost (i.e. 70–90, 130–160 cm depth) at four sites along the China-Russia crude oil pipeline. In the present study we focused on the upper active layer (40 cm depth) where communities of degraders are expected to be more active. In addition, we compiled all pyrosequencing datasets and processed them using a novel statistical and network analysis-based toolkit to obtain information also on habitat preferences and co-occurrence patterns. In this way we could identify specific bacterial interactions that possibly drive hydrocarbon degradation, carbon cycling and the community’s overall functionality in nature. Overall this study, by presenting a conclusive workflow to analyse large-scale environmental sequencing data, moves forward from a standard phylogenetic inventory and advances greatly our understanding of the relationship between biodiversity and ecosystem functioning. In specific this is one of the first contributions to the field of microbial ecology of hydrocarbon contaminated permafrost, and our findings on the evolution of degraders communities provides scientific support to the future development of site-specific bioremediation treatments in cold regions.

## Results

### Microbial cell counts

Microbial cells in uncontaminated permafrost soil samples were in the order of 10^7^–10^8^ per gram of soil (Supplementary Fig. S1). When microcosms were contaminated with crude oil, cell numbers decreased steadily over time in a similar pattern at both low (30%) and high (50%) oil load. After 2 weeks of incubation, cells were reduced approximately by two orders of magnitude and after 8 weeks, at the end of the experiment, cells were in the range of 10^3^–10^5^ cells/g (Supplementary Fig. S1).

### Bacterial community composition in uncontaminated and crude oil contaminated permafrost

Changes in the bacterial communities consequent to crude oil contamination were studied based on pyrosequencing of the DNA extracted from both uncontaminated and contaminated permafrost microcosms. From the 12 samples (4 uncontaminated, 4 at 30% crude oil contamination and 4 at 50% crude oil contamination), a total of valid 75828 reads (21915 from uncontaminated samples, 28043 from 30% crude oil contaminated samples and 25870 from 50% crude oil contaminated samples) was obtained. The rarefaction curves at 3% cutoff (Supplementary Fig. S2) indicated that the bacterial diversity decreased as effect to the exposure to crude oil when compared to the uncontaminated natural samples. The same result, i.e. decrease of OTU richness consequent to contamination with crude oil, was also supported by the estimated indices of ACE, Chao and Shannon (Supplementary Table S1).

**Figure 1:**
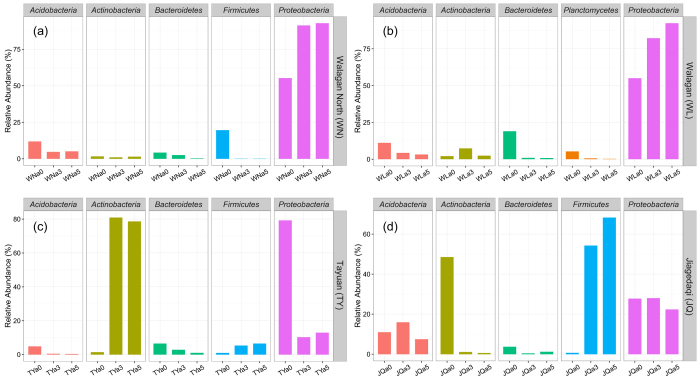
Top 5 most abundant bacterial phyla in samples of uncontaminated and crude oil contaminated permafrost active layer at different study sites. Study sites: (**a**) Walagan North (WN); (**b**) Walagan (WL); (**c**) Tayuan (TY); (**d**) Jiagedaqi (JQ). Following each label (WN, WL, TY and JQ) is the tag “a0, 3, 5” indicating active layer of permafrost (a), uncontaminated (0) and contaminated with 30% (v/w) (3) or 50% v/w (5) crude oil.

**Figure 2:**
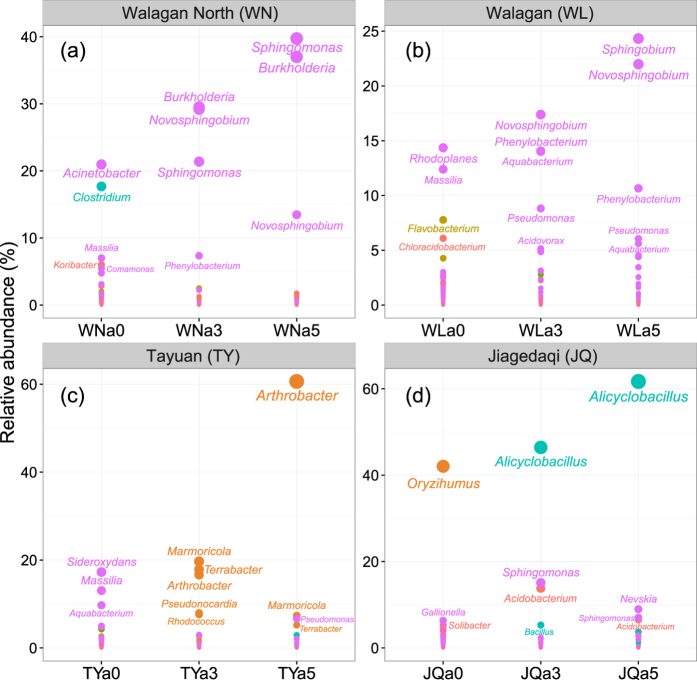
Dominant groups at the genus level in the bacterial communities in samples of uncontaminated and crude oil contaminated permafrost active layers at different study sites. Study sites: (**a**) Walagan North (WN); (**b**) Walagan (WL); (**c**) Tayuan (TY); (**d**) Jiagedaqi (JQ). Following each label (WN, WL, TY and JQ) is the tag “a0, 3, 5” indicating active layer of permafrost (a), uncontaminated (0) and contaminated with 30% (v/w) (3) or 50% v/w (5) crude oil. The size of the dots is proportional to the relative abundance of each bacterial genus. Genus labels are shown only for groups with average abundance above 5%. The same colour is used for genera belonging to same phylum.

Changes of phylotypes abundance induced by crude oil were analysed at the phylum (Fig. 1), family (Supplementary Fig. S3) and genus (Fig. 2) level by comparison to the natural communities. In the Walagan North uncontaminated soil, the natural bacterial community (WNa0) was composed, at the phylum level, mainly of *Proteobacteria* (55.19%), *Firmicutes* (19.59%), *Acidobacteria* (11.84%) and *Bacteroidetes* (4.25%) (Fig. 1a). At the family level (Supplementary Fig. S3a), *Moraxellaceae* (21.44%) was the most abundant and mainly belonging to the genus *Acinetobacter* (20.96%) (Fig. 2a). *Clostridiaceae* (17.68%) were also present with the *Clostridium* genus (17.68%) (Fig. 2a). Following contamination with crude oil, *Proteobacteria* increased further in abundance up to represent about 90% (WNa3: 91.28%; WNa5: 92.77%) of the entire population at both crude oil concentrations (Fig. 1a). In particular, *Sphingomonadaceae* (WNa3: 50.57%; WNa5: 53.18%), *Burkholderiaceae* (WNa3: 30.01%; WNa5: 37.19%) and *Caulobacteraceae* (WNa3: 7.35%; WNa5: 0.60%), were the dominant families (Supplementary Fig. S3a), with *Burkholderia* (WNa3: 29.51%; WNa5: 36.99%), *Novosphingobium* (WNa3: 29.20%; WNa5: 13.46%) and *Sphingomonas* (WNa3: 21.38%; WNa5: 39.73%) (Fig. 2a) being the most abundant genera.

A very similar bacterial profile and response to crude oil was found in the Walagan soil. *Proteobacteria* strongly dominated the community in both uncontaminated (WLa0: 55.04%) and contaminated soil (WLa3: 82.27%; WLa5: 92.36%) (Fig. 1b). Other minor phyla were detected in the natural soil, i.e. *Bacteroidetes* (18.98%), *Acidobacteria* (11.06%) and *Actinobacteria* (2.10%), and all decreased in abundance after crude oil amendment (Fig. 1b). At the family level, the natural community was composed predominantly of *Hyphomicrobiaceae* (18.27%), *Oxalobacteraceae* (13.38%) and *Flavobacteriaceae* (12.04%) (Supplementary Fig. S3b). After crude oil contamination at both 30% load (WLa3) and 50% load (WLa5), the proteobacteria *Sphingomonadaceae* (WLa3: 19.20%; WLa5: 47.04%), *Caulobacteraceae* (WLa3: 19.15%; WLa5: 15.18%), *Comamonadaceae* (WLa3: 22.73%; WLa5: 14.14%) and *Pseudomonadaceae* (WLa3: 8.82%; WLa5: 6.06%) became the dominant groups (Supplementary Fig. S3b). *Novosphingobium* was an abundant member of the communities of contaminated soils (WLa3: 17.39%; WLa5: 21.99%) together with *Sphingobium* (WLa5: 24.32%), *Phenylobacterium* (WLa3: 14.06%; WLa5: 10.66%) and *Pseudomonas* (WLa3: 8.82%; WLa5: 6.06%) (Fig. 2b).

The samples from Tayuan site showed a rather distinctive shift of the bacterial community profile in response to crude oil contamination. Similarly to the other uncontaminated soils, the native bacterial community of TY was dominated by *Proteobacteria* (TYa0: 79.20%) (Fig. 1c) of the families *Gallionellaceae* (20.08%), *Oxalobacteraceae* (18.92%) and *Sphingomonadaceae* (5.10%) (Supplementary Fig. S3c). After contamination with crude oil at both loads (TYa3 and TYa5), *Proteobacteria* decreased significantly to represent only about 10%, while *Actinobacteria* increased to about 80% (80.91% and 78.65% for TYa3 and TYa5, respectively) (Fig. 1c). *Micrococcaceae* (TYa5: 60.66%) and *Nocardioidaceae* (TYa3: 22.62%) became the most abundant members of the community in the contaminated soils, with other minor families identified as *Intrasporangiaceae* (TYa3: 17.91%; TYa5: 5.55%) and *Pseudonocardiaceae* (TYa3: 7.94%) (Supplementary Fig. S3c). At the genus level, *Arthrobacter* (TYa3: 16.65%; TYa5: 60.64%) and *Marmoricola* (TYa3: 19.68%; TYa5: 7.36%) were most abundant but they were hardly detected (<0.5%) in the uncontaminated soils (Fig. 2c).

The bacterial community inhabiting Jiagedaqi natural soil (JQa0) comprised *Actinobacteria* (48.51%), *Proteobacteria* (27.77%) and *Acidobacteria* (10.98%), with *Bacteroidetes* (3.72%) and *Firmicutes* (0.66%) only in traces (Fig. 1d). *Actinobacteria* were mainly affiliated to the family *Intrasporangiaceae* (42.06%) (Supplementary Fig. S3d) and to the genus *Oryzihumus* (42.05%) (Fig. 2d). As clear effect of the addition of crude oil to the soil, *Actinobacteria* decreased substantially to nearly disappear (JQa3: 1.08%; JQa5: 0.60%) while *Firmicutes* increased in abundance up to 54.25% (JQa3) and 68.26% (JQa5) at low and high contamination load, respectively. *Proteobacteria* did not change much in abundance, accounting for approximately 22–28% (JQa3: 27.99%; JQa5: 22.35%) of the community in the contaminated samples. Overall, oil contaminated soils resulted dominated by *Firmicutes* together with *Proteobacteria* (Fig. 1d). This was shown at the family and genus level, where *Alicyclobacillaceae/Alicyclobacillus* (JQa3: 46.86%; JQa5: 62.29% and JQa3: 46.42%; JQa5: 61.69% respectively) were dominant together with *Sphingomonadaceae/Sphingomonas* (JQa3: 17.38%; JQa5: 7.06% and JQa3: 15.11%, JQa5: 7.05% respectively) (Supplementary Fig. S3d; Fig. 2).

The hierarchical heatmap of the top 50 abundant bacterial genera in both uncontaminated and contaminated soils showed the presence of two main clusters (see Supplementary Fig. S4). One cluster, mapping on the right side of the heatmap, comprised mainly the uncontaminated natural soil (WNa0, WLa0 and TYa0). The other cluster included the soil samples treated with crude oil and could be further divided in two subgroups based on the sampling location. The heatmap also showed that amongst the dominant genera, many such as *Novosphingobium*, *Sphingomonas*, *Arthrobacter*, *Burkholderia*, *Phenylobacterium* and *Pseudomonas* are known potent hydrocarbon-degraders (Supplementary Table S2). A number of other minor groups had little, asymmetric or irregular variations in the relative abundance.

### Statistical evaluation of the impact of crude oil contamination and environmental factors on the microbial community composition

Statistical analyses confirmed our findings and observations about shifts in microbial community structure. Analysis of variance using Bray-Curtis distance matrices indicated that the bacterial populations in natural soils and in crude oil contaminated soils were significantly different (*p* = 0.02). The principal coordinates analysis (PCoA) also supported this result, showing that oil contaminated samples in ordination space mapped away from the uncontaminated samples, which grouped close at the bottom left part of the plot. Furthermore, contaminated samples tended to cluster by geographic locations rather than crude oil load (Supplementary Fig. S5).

The multivariate analysis of variance with the adonis function showed that the bacterial communities in the four locations (or sampling sites) were statistically different (*p* < 0.01). It also indicated that the communities established in response to crude oil contamination were significantly different (*p* < 0.01) compared to the natural microbial populations in uncontaminated soil. However different contamination loads (low-30% and high-50%) did not have a major contribution (*p* = 0.76).

### Habitat generalists vs. specialists

Once established that oil contamination causes significant changes in the bacterial biodiversity of affected permafrost soil, we set out to explore the ecological and functional role of representative taxa. To work on a larger and more comprehensive dataset, we compiled the pyrosequencing data from the present study (uncontaminated and contaminated upper active layer, 30–40 cm depth) with data from previous studies done in parallel (uncontaminated and contaminated deeper permafrost, 70–90 cm depth)^15^,^22^. We analysed which taxa are generalists (i.e. broadly distributed) or specialists (i.e. specifically distributed) in relation to the habitat, taking in consideration not only the uncontaminated/contaminated permafrost but also the permafrost depth i.e. upper/deeper layers.

The group of specialists for contaminated permafrost comprised 51.60% of the total OTUs (89682 out of 173769), while specialists for uncontaminated were 36.29% (63060 out of 173769), generalists were 11.33% (19705 out of 173769) and only 0.76% (1322 out of 173769) were the taxa too rare to be classified. With respect to permafrost depth, distribution of specialists for upper (27.15%, i.e. 47178 out of 173769) and deeper layer (26.16%, i.e. 45458 out of 173769) was comparable, while the generalists accounted for 45.95% (79847 out of 173769) and the rare taxa for 0.74% (1286 out of 173769). These data further confirmed that crude oil contamination clearly leads to the development of a locally abundant and specialised community of bacterial taxa.

### Crude oil contaminated permafrost: specialists and network analysis

**Figure 3:**
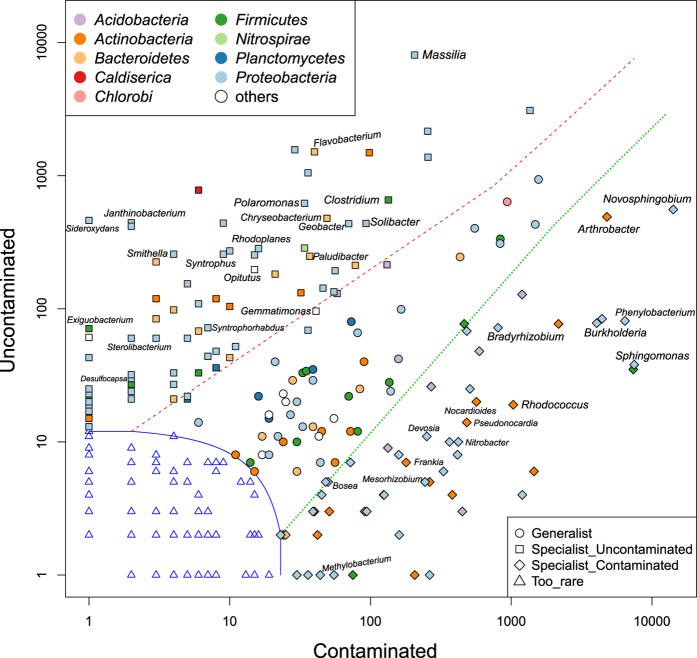
Habitat generalists and specialists in relation to uncontaminated and crude oil contaminated permafrost samples. The classification of generalists and specialists was done based on the function CLAM test in vegan package according to the estimated species relative abundance in uncontaminated and contaminated samples. The test was applied with arguments of default alpha and a specialisation threshold of 2/3 according to the supermajority rule. The x and y axes represent the abundance of different genera in contaminated and uncontaminated samples respectively. All the counts were added by 1 to let the marginal taxa evenly arranged in the plot space. Specialists for uncontaminated soil map at the left side of the plot, specialists for contaminated soil at the right side, generalists are located in the middle and rare taxa are at the bottom left corner. Labels are only shown for representative specialists used for co-occurrence patterns and network analysis.

**Figure 4:**
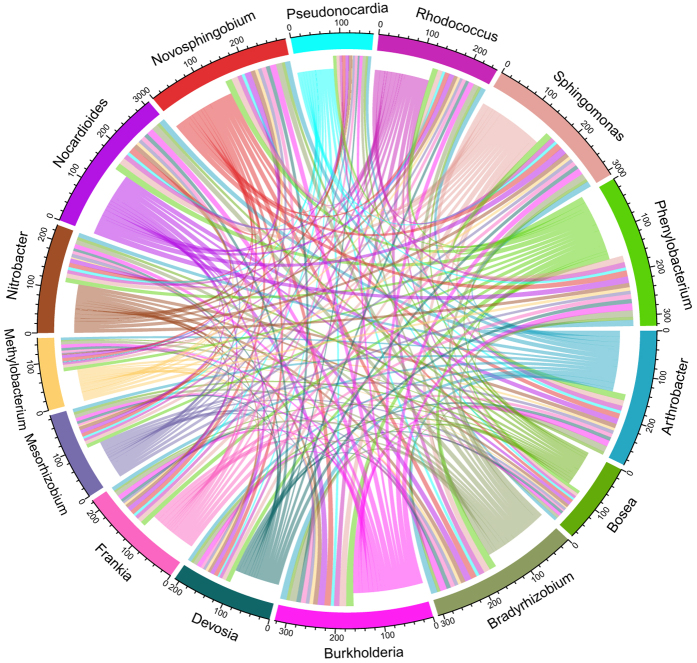
Chord diagram displaying the network of selected (15) co-occurring specialists in crude oil contaminated permafrost samples. Each sector of the circle represents one node (i.e. taxon) of the network, and its width indicates the total amount of co-occurrence that connects a certain bacterial taxon A (e.g. *Burkholderia*) to the other taxa, together with the total amount of co-occurrence that connects all the other taxa to bacterial taxon A. The width of each link represents the total co-occurrence of e.g. bacterial taxon A (e.g. *Burkholderia*) with taxon B (e.g. *Phenylobacterium*) specifically, i.e. the total number of samples in which the co-occurring taxa pairwise is detected. The Chord diagram was generated using the circlize package in *R* based on the adjacency matrix for the genera of interest.

Examined in more detail, we found that amongst the 47 taxa specialists for contaminated permafrost (Fig. 3), 15 included hydrocarbon degrader genera with typical distribution in either the bulk soil, e.g. *Novosphingobium* (15.45% relative abundance), *Sphingomonas* (10.11%), *Phenylobacterium* (10.07%), *Arthrobacter* (6.33%), *Burkholderia* (5.40%), *Rhodococcus* (1.56%), *Nocardioides* (0.66%) and *Pseudonocardia* (0.56%), or in the rhizosphere, e.g. *Bradyrhizobium* (1.01%), *Mesorhizobium* (0.27%), *Nitrobacter* (0.46%), *Frankia* (0.28%), *Devosia* (0.26%), *Methylobacterium* (0.07%) and *Bosea* (0.05%). Based on the CLAM test, degrader specialists associated with bulk soil were equally distributed along the permafrost depth (upper layer specialists 3/8; deep layer specialists 3/8; generalists 2/8), whereas those associated to the rhizosphere were mostly generalists (generalists 5/7; deep layer specialists 2/7) (Supplementary Fig. S6). Furthermore, to identify potential interactions between community members, we performed network analysis on the 15 selected specialists. We first quantified the tendency of two nodes to co-occur (i.e. pairwise non-random co-occurrence), which indicates that two taxa are more closely related than it would be expected to occur randomly. As a positive relationship is assumed on the basis of co-occurrence, this test allows also to predict interactions such as co-metabolism, co-aggregation, co-colonisation and niche overlap. We also analysed the node degree distribution, which quantifies the number of connections each node (i.e. each taxon) has to other nodes in the network. Since the microbial community under investigation was rather complex and with numerous possible connections amongst members, we opted to perform the degree distribution analysis on the upper half (i.e. all nodes with weighted edges above the median value) of the most frequently co-occurring pairs, which allowed us to bring into focus the most meaningful taxa and their interactions within the network. We found that all taxa were connected with each other but that *Burkholderia* was certainly a keystone taxon within the network, forming the most frequently co-occurring pairwise with the other members, and particularly with *Phenylobacterium*, *Sphingomonas*, *Nocardioides* and *Novosphingobium* (Fig. 4; Table 1). We observed that also members of the *Rhizobiales* order, and to the highest degree *Bradyrhizobium*, strongly co-occurred with other hydrocarbon-degrading organisms, mainly *Burkholderia*, *Arthrobacter* and *Rhodococcus* (Fig. 4; Table 1). Our results seem to suggest that: 1) cooperative interactions amongst different bacterial species take place in order to accomplish the degradation of crude oil and polycyclic aromatic hydrocarbons (PAHs) in particular; 2) *Phenylobacterium*, even if it is not (yet) a well-studied organism for hydrocarbon metabolism, appears to be a new and important player within the network of known degraders; 3) rhizosphere-associated microorganisms are also involved in the hydrocarbon degradation processes.

### Uncontaminated permafrost: specialists and network analysis

Amongst the specialists of the uncontaminated permafrost (Fig. 3) were present taxa whose members are typical aerobic soil microorganisms often found in cold-environments such as *Massilia* (18% relative abundance), *Pseudomonas* (6.08%), *Flavobacterium* (3.42%), *Solibacter* (1.78%), *Polaromonas* (1.46%), *Chryseobacterium* (1.28%), *Janthinobacterium* (0.95%) and *Exiguobacterium* (0.15%). However, no distinct co-occurrence network could be identified for these cold-adapted taxa.

Within the specialists of uncontaminated permafrost we recognised also several (i.e. 12) taxa potentially involved in the anaerobic cycling of carbon in soil, e.g. *Geobacter* (1.87%), *Clostridium* (1.79%), *Sideroxydans* (1.51%), *Rhodoplanes* (1.46%), *Opitutus* (0.79%), *Syntrophus* (0.76%), *Smithella* (0.74%), *Paludibacter* (0.62%), *Gemmatimonas* (0.39%), *Syntrophorhabdus* (0.26%), *Sterolibacterium* (0.18%) and *Desulfocapsa* (0.09%).

**Figure 5:**
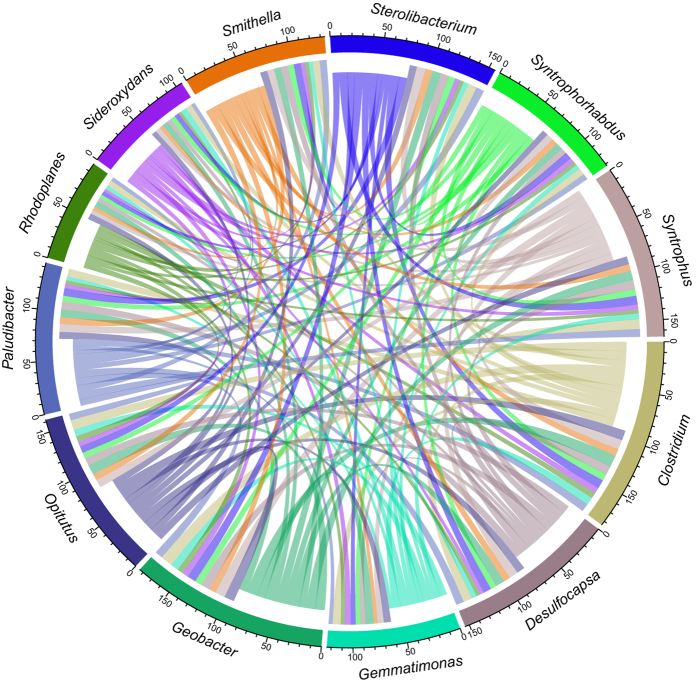
Chord diagram displaying the network of selected (12) co-occurring specialists in uncontaminated permafrost samples. Each sector of the circle represents one node (i.e. taxon) of the network, and its width indicates the total amount of co-occurrence that connects a certain bacterial taxon A (e.g. *Clostridium*) to the other taxa, together with the total amount of co-occurrence that connects all the other taxa to bacterial taxon A. The width of each link represents the total co-occurrence of e.g. bacterial taxon A (e.g. *Clostridium*) with taxon B (e.g. *Geobacter*) specifically, i.e. the total number of samples in which the co-occurring taxa pairwise is detected. The Chord diagram was generated using the circlize package in *R* based on the adjacency matrix for the genera of interest.

To investigate possible interactions and co-occurrence patterns within the 12 selected taxa, we followed the same approach based on network analysis applied previously. First, we used the CLAM test to study the habitat distribution and found that most taxa are generalists with regards to upper/deeper layer (Supplementary Fig. S6), which indicates that 1) they are broadly distributed along the permafrost depth and that 2) they share the same habitat. We then investigated further the possible interactions between the selected taxa based on pairwise co-occurrence and node degree distribution. All taxa were found to have connections with each other but *Clostridium* clearly represented a keystone taxon within the network, with tendency to co-occur in particular together with *Geobacter*, *Syntrophus*, *Opitutus* and *Sterolibacter* (Fig. 5; Table 2). These findings seem to indicate the presence of a syntrophic microbial network based on anaerobic carbon degradation. *Clostridium* is so highly interconnected with the other taxa because, as a primary producer, it feeds in short chain fatty acids (e.g. acetate, butyrate, propionate) and hydrogen that are used as growth substrate by the other members of the network. This is the case, for example, of the pair *Clostridium*/*Geobacter*, the most frequently and strongly co-occurring, where *Geobacter* is likely to utilise acetate (and other fatty acids) as electron donors.

## Discussion

In the first part of this work we applied 16 S rRNA deep amplicon sequencing to examine the bacterial biodiversity and community structure in permafrost upper active layer located in an area crossed by the large China-Russia crude oil pipeline (CRCOP) and how they change in response to crude oil contamination in the event of an accidental oil spill. Our first observation was that, consequent to contamination, there was a clear decrease of bacterial cell numbers and reduction of the species diversity. This has been shown as a common trend in microbial populations exposed to hydrocarbons and has been demonstrated not only based on 16 S rRNA genes^23^,^24^ but also in relation to functional genes for hydrocarbon degradation such as alkane monooxigenase, cytochrome P450, alcohol dehydrogenase and aldehyde dehydrogenase^24^. Reduced species richness is caused by the selective ecological pressure of hydrocarbon contamination, which involves: (i) the toxic effect of hydrocarbons towards microbial cells; (ii) the abundance of hydrocarbon-derived substrates over the typical soil organic matter compounds; (iii) the imbalance of the C:N:P ratios due to an excessive input of carbon compared to the available micronutrients^23^.

In addition, selective pressure favours the enrichment of certain species that are physiologically and metabolically adapted to hydrocarbons, which leads to shifts in the dominant phylotypes and community‘s overall structure. Reduced biodiversity accompanied with dominance of few selected species in contaminated environments has been previously shown to enhance bioremediation^24^,^25^. This seems to be based on the principle that increasing environmental disturbance favours interspecies competition^26^. Our second observation is in line with these considerations. In the Walagan (WL) and Walagan North (WN) sites, *Proteobacteria*, already the most abundant in the pristine soil, were enriched further in the contaminated microcosms. The major families, i.e. *Sphingomonadaceae* and *Burkholderiaceae*, are known to possess large plasmids (or mega-plasmids) that confer an extraordinary catabolic versatility and resistance, and contribute to the capability to degrade a wide range of hydrocarbons, alkanes and mono- and polycyclic aromatic compounds^27^,^28^. Sphingomonads, in particular, have been previously described as key players in hydrocarbon degrading communities in cold soils^4^,^7^,^14^. *Caulobacteriaceae* were also enriched upon contamination and, although not amongst the typical hydrocarbon degrading organisms, they have been increasingly detected in contaminated cold soils especially via gene-based analyses^4^,^14^,^29^. A quite different response was instead observed in the Tayuan (TY) site where exposure to crude oil caused a distinct shift from *Proteobacteria*- to *Actinobacteria*-dominated bacterial community. This was mainly due to an increase in abundance of *Arthrobacter*, a known hydrocarbon degrader widespread in cold contaminated environments^11^,^30^. Recent works of genome sequencing have provided important data to understand the genetic basis of hydrocarbon and xenobiotic metabolism of *Arthrobacter* species. For example, 87 genes could be annotated in the genome of *Arthrobacter* sp. YC-RL1 as involved in the degradation of a large variety of hydrocarbons including polycyclic aromatics, toluene, xylene, ethylbenzene and other chemicals^31^. Similar results with bacterial communities enriched in *Proteobacteria* and *Actinobacteria* were reported recently for diesel-contaminated soil microcosms^24^. Differently from the other study sites, crude oil contamination of Jiagedaqi (JQ) soil resulted in the bacterial community to enrich remarkably in *Firmicutes*, mostly of the *Alicyclobacillus* genus. *Alicyclobacillus* comprise spore-forming bacteria, typically thermo-acidophilic but capable to grow also at around 20 °C. Although their capability to degrade hydrocarbons is largely unknown, a few works have reported their presence and potential oxidising-activity in crude oil reservoirs^32^,^33^.

All together these findings indicate that the response of bacterial communities to the environmental perturbation with hydrocarbons is highly site-specific and that various organisms with degradative capabilities can become dominant phylotypes. In particular, a trend of decreasing bacterial community similarity with increasing sampling site distance could be observed (i.e. Walagan and Walagan North being the closest and most similar, and Jiagedaqi being the most distant and diverse), which can be explained by increasing differences in environmental factors. Other studies have highlighted the primary role of environmental properties (e.g. geographical location, soil physico-chemical properties and vegetation besides oil contamination) in shaping the communities of hydrocarbon degrading microorganisms at a certain site^34^,^35^. Therefore, there is growing evidence that bioremediation treatments should be designed as site-specific and based on a profound knowledge of the local microbial communities, their initial biodiversity and structure, and how they develop in response to the exposure to contaminants.

In any ecological niche microorganisms form complex and highly specific networks of interactions^36^. In the second part of the study, our objectives were to identify specialists and generalists taxa, non-random co-occurrence patterns and connections amongst them with the aim to understand better what keystone taxa may be present within the community, how they interact and what bioprocesses may drive in the natural environment. To do so, we applied network analysis to a larger environmental dataset integrating the pyrosequencing data from this and parallel works^15^,^22^ that investigated the microbial diversity of permafrost along the China-Russia crude oil pipeline.

Biodegradation processes in nature encompass complex metabolic and regulatory interactions that modern high-throughput techniques such as metagenomics can help accessing^37^,^38^. Previous studies have shown that metagenomes can be predictive of the metabolic potential within an ecosystem^5^. In our network, the 15 nodes representing taxa specialists for soils contaminated with crude oil showed high connectivity with each other implying that cooperative interactions may be present also in nature. Most keystone taxa belonged to known hydrocarbon-degrading species, e.g. *Sphingomonas*, *Novosphingobium*, *Burkholderia*, *Arthrobacter*, *Rhodococcus* and *Nocardia*, which experimental data has often demonstrated to co-occur in consortia^39^,^40^,^41^. Especially in the case of complex mixtures of hydrocarbons and PAHs, cooperative interactions (e.g. co-metabolism, synthesis of biosurfactants, adhesion via biofilm), deriving from the synergetic activity of a number of bacterial species, are known to enhance significantly the degradation rates^42^,^43^. *Phenylobacterium*, a taxon with high number of connections in our network, is not a well-studied genus at present, however sequences related to this bacterium have been detected in soils contaminated with crude oil^35^, PAHs^41^, polychlorinated biphenyl (PCB)^44^. The sequencing of the genome of *Phenylobacterium immobile* strain E (DSM 1986) has recently revealed the presence of a high number of Rieske non-heme iron aromatic ring-hydroxylating oxygenases (RHOs), a large family of multi-component enzymes involved in the initial step of degradation of aromatic compounds, which makes them enzymatically accessible for further degradation by other organisms^45^. This may well explain the high connectivity of *Phenylobacterium* with other hydrocarbon-degraders that we observed in our network analysis.

Furthermore, many nodes in the network of specialists for contaminated soil belonged to the *Rhizobiales*. This genus comprises known plant symbionts and nitrogen-fixing bacteria, and more recently some have been reported as capable to degrade PAHs^46^,^47^. Furthermore, genomic analysis showed that some species of *Rhizobium*, *Bradyrhizobium* and *Mesorhizobium* additionally harbour multiple genes for acyl-homoserine lactone (AHL) or autoinducer (AI) quorum-sensing (QS) molecules, which may suggest that bacterial hydrocarbon degradation is a QS-regulated process^46^,^48^. Several studies have demonstrated that degradation of environmental contaminants is enhanced in the plant rhizosphere^47^,^49^ and inoculation with *Rhizobium melitoti* was reported to specifically stimulate the growth and activity of indigenous hydrocarbon-degrading microorganisms^50^. In the light of these observations, our data further support the concept that increased selection pressure due to exposure to contaminants stimulates communication and cooperation amongst members of the permafrost microbial community, especially in the upper active layer.

In absence of the pressure exerted by crude oil contamination, the most consolidated network of taxa in permafrost was found to comprise anaerobic bacteria, presumably pertaining the low-oxygen deeper layer, linked by metabolic and synthrophic interactions. Overall, the network we described included: primary fermentative bacteria capable to degrade plant polymers and dead microbial biomass (e.g. *Clostridium*, *Sterolibacterium*, *Opitutus* and *Paludibacter*) with consequent production of short chain fatty acids and alcohols together with CO_2_ and H_2_; secondary fermentative bacteria (e.g. *Syntrophus*, *Smithella* and *Syntrophorhabdus*) capable to oxide intermediate products (e.g. propionate and benzoate) with release of acetate and H_2_; sulfate reducers (e.g. *Desulfocapsa*) and metal reducers (e.g. *Geobacter*). Most of these microorganisms are known syntrophs, i.e. they operate in cooperation with a second, complementary, microorganism that consumes their metabolic end-products with high affinity, thus enabling a metabolic reaction, which could not be possible by either organisms alone, to proceed under thermodynamically favourable conditions^51^,^52^. Syntrophic associations typically involve hydrogen- or acetate-consuming methanogenic archaea that perform the last step of the anaerobic carbon cycle by releasing methane. Although our study did not target this specific group, it is likely that methanogens were present in our samples and would have been part of the network. Other studies have described similar communities in analogous environments, i.e. the Arctic permafrost^53^,^54^ and a methane-emitting wetland on Spruce Mountains^55^, which poses these bacterial OTUs as keystones within the carbon cycle network. Members of the family *Syntrophaceae* (*Synthrophus* and *Smithella*) have been additionally identified in hydrocarbon-impacted environments, implying that they may be players also in syntrophic hydrocarbon metabolism^56^.

Co-metabolism and syntrophy are essential in nature for the complete conversion of soil organic material to CO_2_ and CH_4_^51^. However our understanding of ecosystem-functionally relevant species is hampered by the difficulty to cultivate the interacting organisms and characterise their eco-physiological aspects in the laboratory. Meta-omics approaches (e.g. meta-genomics, transcriptomics, proteomics) integrated with a deep analysis of the large datasets generated, as it is possible for example with network analysis, offers a valid help disentangling the complexity of microbial and trophic interactions that occur in the environment. Here we made use of this approach to highlight that the keystone taxa of permafrost affected sites show large differences prior to and after exposure to crude oil in the way that networks of syntrophic carbon cycling bacteria are replaced by hydrocarbon degraders. Crude oil contamination thus has severe effects on the indigenous bacterial community in permafrost environments and allows for a rapid establishment of typical hydrocarbon degraders. The knowledge of endemism and trophic correlations of bacterial degraders will be helpful in developing biodegradation treatments in nature.

## Methods

### Research Sites

Permafrost samples were collected as described by Yang *et al*.^22^ from four different study sites, Walagan North (WN), Walagan (WL), Tayuan (TY) and Jiagedaqi (JQ), covering a total distance of about 300 km along the China-Russia crude oil pipeline (CRCOP) (Supplementary Table S3). These sites were selected as previously identified as of high risk of oil spills^2^. A detailed analysis of site-specific environmental characteristics including climate and ecosystems can be found in Yang *et al*.^2^. Samples consisted of soil from the upper active layer (20–40 cm) of permafrost located in an acidic bog-type wetland area. Temperature in this region may be extreme, ranging from −40 °C to 40 °C, although on average it is −26 °C in winter time and 19 °C in summer (Supplementary Table S1).

### Microcosm preparation and incubation

Microcosms were prepared by weighing 10 g of permafrost into a 50 ml glass jar and adding the crude oil transported in the CRCOP pipeline at two different concentrations (30% and 50% v/w). To allow abiotic loss via evaporation of the volatile fraction of hydrocarbons, oil-contaminated soil was let to weather incubated at 1 °C for 1 week. Uncontaminated soils, as controls, were equilibrated in the same way. After equilibration, microcosms were incubated at 20 °C for 8 weeks. Duration and temperature of the experiment were selected to simulate the optimal environmental conditions on site during the summer time from July to August. All throughout the incubation period, microcosms were constantly aerated by supplying oxygen via a U-shaped tube equipped with sterile filter to remove air contaminants. For each contamination load (uncontaminated, 30% v/w oil-contaminated and 50% v/w oil-contaminated) and incubation time (0, 2, 4, 6 and 8 weeks), microcosms were prepared in triplicates.

### Total cell enumeration

At 2 week-time intervals, one set of microcosms was sacrificed and used for cell enumeration. Total microbial cells were counted as follows. Briefly, 2 g of soil sample were suspended in 10 ml TE buffer (10 mM Tris-HCl, 1 M EDTA; pH 7.5), centrifuged at 6000 × *g* for 5 min, and 2 ml of clean supernatant (carefully collected from underneath the top oil layer) were transferred to a fresh tube. Cells present in the liquid were fixed with 3% (v/v) formaldehyde solution, collected by filtration onto a nitrocellulose membrane (0.22 μm, Whatman) and finally stained with 2.5× SYBR Green I (Molecular Probes, Invitrogen) for 15 min in the dark. Microbial cells were counted using a microscope Olympus BX51 (Olympus, Japan), and cell numbers were calculated based on the counts of at least 10 random eye fields taking into account the sample dilution and soil weight. This assay was done in three biological replicates.

### DNA extraction, PCR and pyrosequencing

DNA was extracted in triplicate using the Power Soil DNA Isolation Kit (MOBIO, USA) according to the manufacturer’s instructions. The bacterial primer set 8 F (5′−3′ GAGTTTGATCCTGGCTCAG) and 533 R (5′−3′ TTACCGCGGCTGCTGGCAC) was used to amplify the V1-V3 regions of 16 S rRNA gene, with barcodes incorporated at the 5′ end of the forward primer. The PCR reaction was performed in a 20 μl-reaction volume containing 0.5 μl DNA template, 250 μM dNTPs, 0.1 μM of each primer, 2.5 U FastPfu Polymerase (Applied Biosystems) and 5× FastPfu buffer to final volume. The PCR program was as follows: 2 min at 95 °C, followed by 25 cycles of 94 °C for 30 s, 55 °C for 30 s and 72 °C for 30 s, with a final extension of 5 min at 72 °C. After purification (MiniElute PCR purification kit, Qiagen), the PCR products were quantified using the GeneQuant *pro* spectrophotometer. The three replicates were pooled together prior to metagenomic sequencing to maximise the depth of sequencing. The equalised PCR products were sequenced using a Roche GS-FLX sequencer (454 Life Sciences) at the Shanghai Majorbio Bio-pharm Technology Co., Ltd.

### Bioinformatic Analysis

Raw sequence data were processed using the series of commands in mothur (version 1.32.1) software environment^57^. The following initial steps were performed: trimming of pyrosequencing tag sequences and primers, and removal of sequences with more than eight homopolymer nucleotides. The sequences were then limited to those longer than 300 bp. The unique sequences were then aligned with the bacterial SILVA database (SILVA 108) using default settings. After chimera checking, low quality sequences were removed. The sequences passing these checks were then used to compute the sequence distance matrix. The operational taxonomic units (OTUs) were classified at identities of 97% and 95%. Rarefaction curve was generated by using the *R* package vegan v.2.0–7 (available at http://CRAN.R-project.org/package=vegan), and the heatmap was constructed with the *R* package of pheatmap v. 0.7.4 (available at http://CRAN.R-project.org/package=pheatmap).

### Statistical Analysis

Permutational multivariate analysis of variance using distance matrices was performed with the adonis function in the vegan package, which partitions sums of squares using semi-metric and metric distance matrices and fit linear models (e.g. factors, polynomial regression) to distance matrices. Furthermore, the multinomial species classification method (CLAM test)^58^ in the vegan *R* package was applied to statistically classify the bacterial OTUs at the genus level based on habitat into the following categories: 1) generalists, 2) specialists for permafrost upper layer, 3) specialists for permafrost deeper layer, 4) too rare to classify with confidence, and also based on crude oil contamination into the following categories: 1) generalists, 2) specialists for uncontaminated permafrost soil, 3) specialists for crude oil-contaminated permafrost soil and 4) too rare to classify with confidence. The CLAM test was performed on the dataset retrieved in this study and compiled with datasets from previous works on the same permafrost habitat^15^,^22^. The CLAM test was applied on subsampled datasets using a default alpha value, a coverage limit of 30 and a specialisation threshold of 0.67 (a specialisation threshold of 0.67, i.e. supermajority, is considered conservative according to Chazdon *et al*.^58^).

### Network Analysis

To reduce rare OTUs in the dataset, OTUs with average relative abundances less than 0.01% were removed from the total number of bacterial sequences. Furthermore, a valid (or direct) co-occurrence event was considered representing a robust correlation if the Spearman’s correlation coefficient (rho) was >0.6 and statistically significant (*p* < 0.01). These two filtering steps were applied to remove poorly represented OTUs and reduce network complexity, which assisted in determining the bacterial core community of the samples under investigation. Subsequently, the affiliation network was generated by using the *R* package of igraph, version 1.0.1 (available at http://igraph.org). An affiliation network is defined as a network where the members are affiliated with one another based on co-membership in a group or co-participation in some type of event. The direct connections amongst the different genera were extracted from two-mode affiliation network using igraph’s bipartite.projection function. The nodes in the reconstructed network represented variable genera and the edges that connect the nodes represented correlations between genera. Network’s topography features including centrality and edge weights were also analysed using functions available in igraph package. In addition, to bring into focus potential keystone taxa and their interactions within the network, subsets of representative specialist genera from both contaminated and uncontaminated samples were selected. Sub-networks were then generated from the meta-community networks by using subgraph functions in igraph package. Information on the target network was further organised in matrices and visualised in chord diagrams using *R* package circlize, version 0.3.5 (available at https://cran.r-project.org/web/packages/circlize/index.html).

### Data Availability

All sequences generated in this work have been deposited at NCBI Sequence Read Archive of SRR548601 and SRR1055249.

## Additional Information

**How to cite this article**: Yang, S. *et al*. Hydrocarbon degraders establish at the costs of microbial richness, abundance and keystone taxa after crude oil contamination in permafrost environments. *Sci. Rep*. **6**, 37473; doi: 10.1038/srep37473 (2016).

**Publisher’s note:** Springer Nature remains neutral with regard to jurisdictional claims in published maps and institutional affiliations.

## Supplementary Material

Supplementary Information

## Figures and Tables

**Table 1:** Pairwise co-occurrence analysis of selected (15) specialists in crude oil contaminated permafrost samples possibly involved in hydrocarbon degradation.

No.	Taxon (i.e. node)	Number of connections^a^	Strongest pairwise co-occurrence^b^ (e-weight^c^)
1	*Burkholderia*	14	*Phenylobacterium* (16)
2	*Phenylobacterium*	14	*Burkholderia* (16)
5	*Sphingomonas*	14	*Burkholderia* (15)
4	*Nocardioides*	14	*Burkholderia* (15)
3	*Bradyrhizobium*	14	*Burkholderia* (15)
7	*Novosphingobium*	14	*Burkholderia* (14)
6	*Arthrobacter*	14	*Bradyrhizobium* (14)
8	*Rhodococcus*	14	*Bradyrhizobium* (12)
9	*Frankia*	14	*Burkholderia* (11)
10	*Devosia*	14	*Burkholderia* (10)
11	*Nitrobacter*	14	*Arthrobacter* (10)
12	*Mesorhizobium*	14	*Arthrobacter* (8)
13	*Methylobacterium*	14	*Burkholderia* (8)
14	*Bosea*	14	*Arthrobacter* (7)
15	*Pseudonocardia*	14	*Arthrobacter* (7)

^a^Total number of other taxa (max 14) with which each taxon co-occurred.

^b^Most frequently co-occurring taxa pairwise.

^c^Total number of samples (max 16) in which the co-occurring pairwise is found.

**Table 2:** Pairwise co-occurrence analysis of selected (12) specialists in uncontaminated permafrost samples possibly involved in anaerobic carbon cycle.

No.	Taxon (i.e. node)	Number of connections^a^	Strongest pairwise co-occurrence^b^ (e-weight^c^)
1	*Clostridium*	11	*Geobacter* (12)
2	*Geobacter*	11	*Clostridium* (12)
3	*Syntrophus*	11	*Clostridium*, *Geobacter* (10)
4	*Opitutus*	11	*Clostridium*, *Geobacter* (10)
5	*Sterolibacterium*	11	*Clostridium*, *Geobacter*, *Syntrophus* (9)
6	*Desulfocapsa*	11	*Clostridium*, *Geobacter*, *Syntrophus* (9)
7	*Gemmatimonas*	11	*Clostridium*, *Geobacter* (8)
8	*Paludibacter*	11	*Clostridium*, *Desulfocapsa*, *Geobacter*, *Sterolibacterium*, *Synthrophus* (8)
9	*Syntrophorhabdus*	11	*Clostridium*, *Geobacter*, *Sterolibacterium*, *Synthrophus* (7)
10	*Smithella*	11	*Clostridium*, *Desulfocapsa*, *Geobacter*, *Opitutus*, *Paludibacter*, *Sterolibacterium*, *Synthrophus* (7)
11	*Sideroxydans*	11	*Clostridium*, *Geobacter*, *Gemmatimonas* (6)
12	*Rhodoplanes*	11	*Clostridium*, *Geobacter*, *Opitutus* (6)

^a^Total number of other taxa (max 11) with which each taxon co-occurred.

^b^Most frequently co-occurring taxa pairwise.

^c^Total number of samples (max 12) in which the co-occurring pairwise is found.
