# Supplementary material for: Hydrocarbon degraders establish at the costs of microbial richness, abundance and keystone taxa after crude oil contamination in permafrost environments
Source: Sci Rep. 2016 Nov 25;6:37473. doi: 10.1038/srep37473 (PMC5122841; doi:10.1038/srep37473)
Supplement: Supplementary Information [file srep37473-s1.pdf]

Supplementary Information

Hydrocarbon degraders establish at the costs of microbial richness, abundance and keystone taxa after crude oil contamination in permafrost environments

Sizhong Yang[1, 2], Xi Wen[2, 3], Yulan Shi[1], Susanne Liebner[2], Huijun Jin[1]*, Amedea Perfumo[2]*

[1] State Key Laboratory of Frozen Soils Engineering (SKLFSE), Northwest Institute of Eco-Environment and Resources, Chinese Academy of Sciences (CAS), Lanzhou, 730000, China;

[2] GFZ German Research Centre for Geosciences, Helmholtz Centre Potsdam, Section 5.3 Geomicrobiology, Telegrafenberg, 14473 Potsdam, Germany;

[3] College of Electrical Engineering, Northwest University for Nationalities, Lanzhou, 730030, China

*Corresponding authors: hjjin@lzb.ac.cn and amedea.perfumo@gfz-potsdam.de

Scientific Reports

The supplementary information includes Fig. S1, Fig. S2, Fig. S3, Fig. S4, Fig. S5, Fig. S6. and Table S1, Table S2, Table S3.

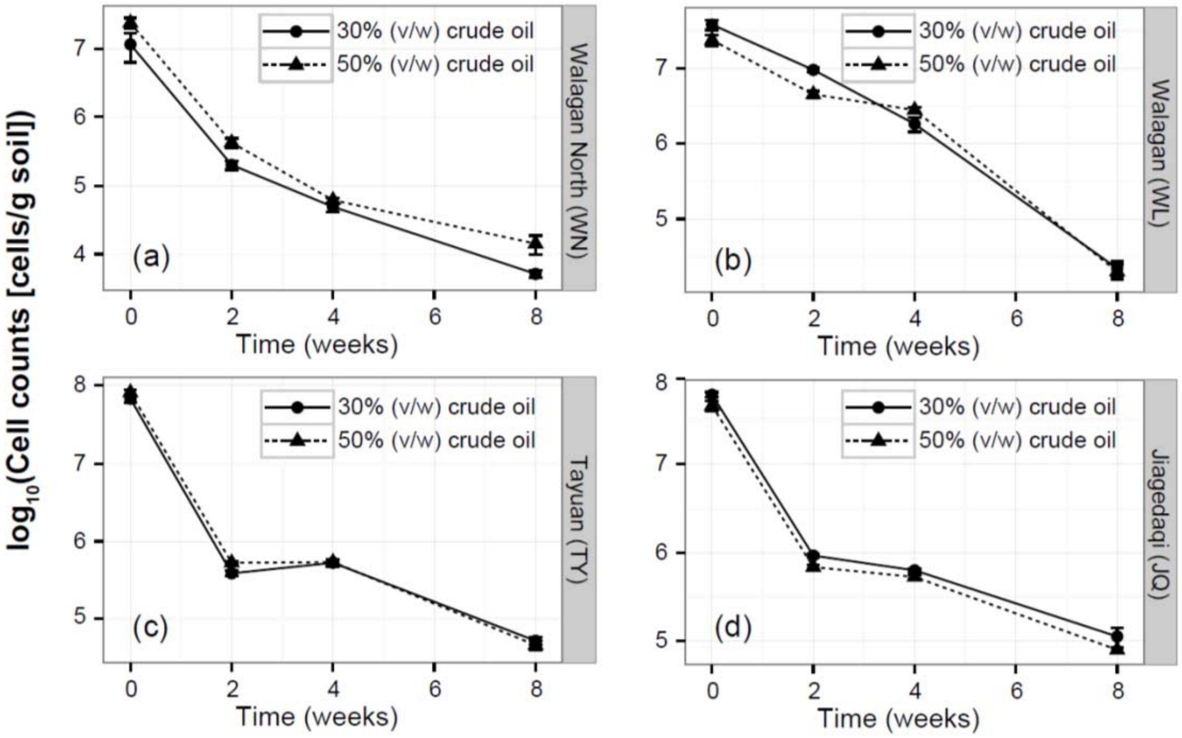

Fig. S1. Time-course microbial cell enumeration in microcosms with permafrost active layer contaminated with crude oil. Study sites are: a) Walagan North (WN); b) Walagan (WL); c) Tayuan (TY); d) Jiagedaqi (JQ). Cell counts were performed in SYBR Green I-stained samples. The shown values are the average of three replicates and error bars indicate standard deviation.

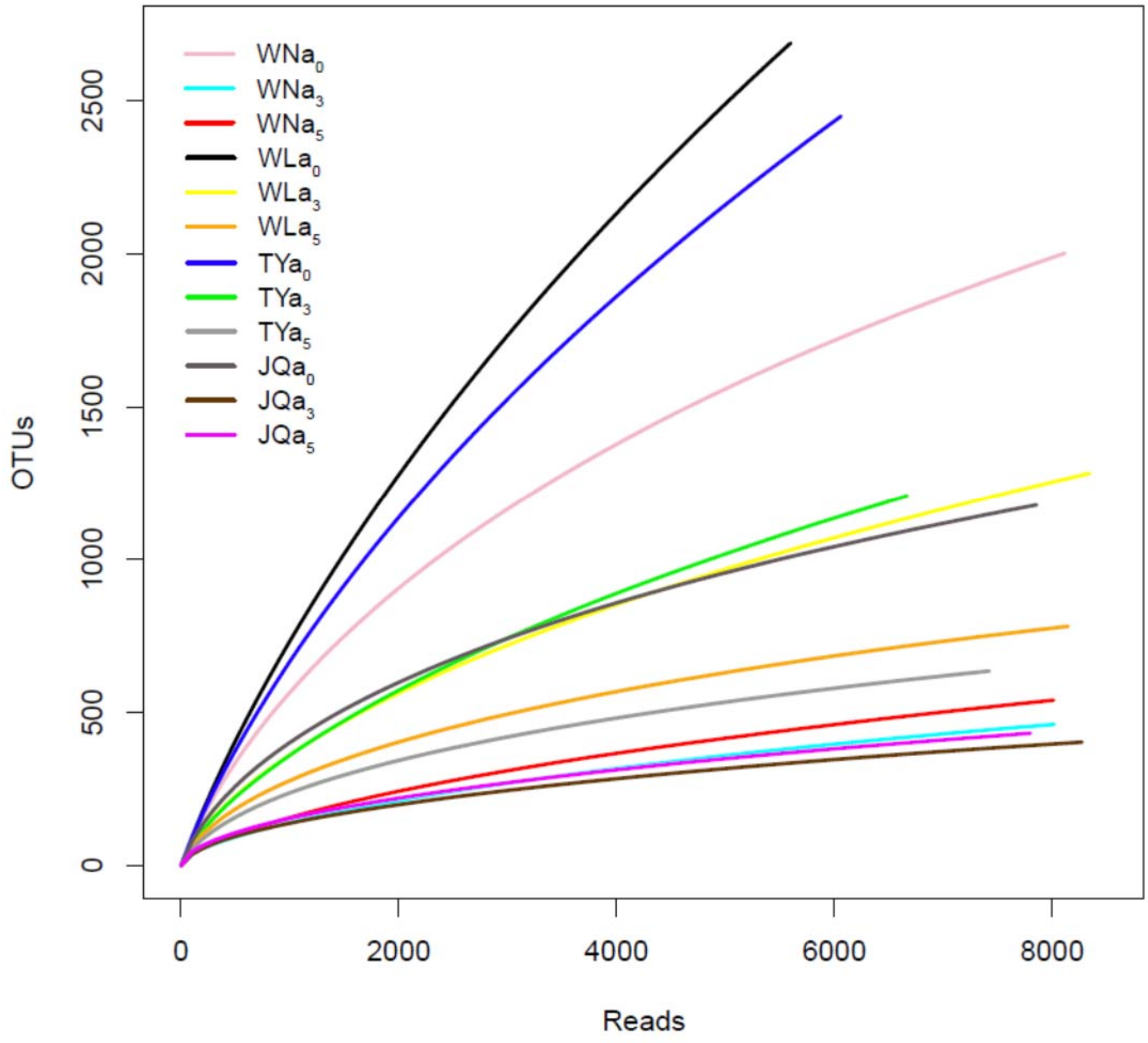

Fig. S2. Rarefaction curves of the observed 97% OTU richness of the bacterial community profiles in samples of uncontaminated and crude oil contaminated permafrost active layer. Study sites are a) Walagan North (WN); b) Walagan (WL); c) Tayuan (TY); d) Jiagedaqi (JQ). Following each label (WN, WL, TY and JQ) is the tag "a0, 3, 5" indicating active layer of permafrost (a), uncontaminated (0) and contaminated with 30% (v/w) (3) or 50% v/w (5) crude oil.

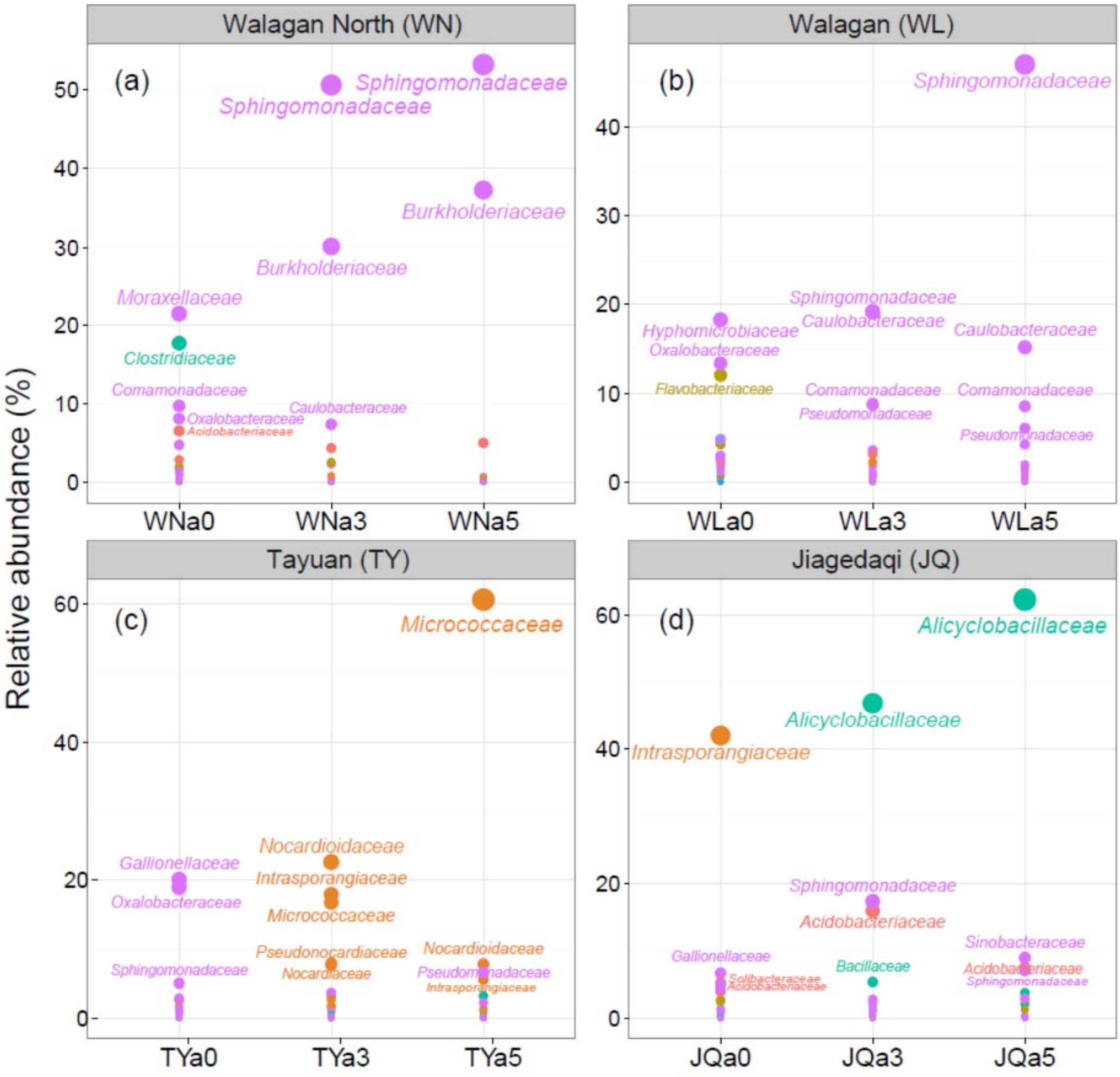

Fig. S3. Dominant groups at the family level in the bacterial communities in samples of uncontaminated and crude oil contaminated permafrost active layer at different study sites. The size of the dots is proportional to the relative abundance of each bacterial family. Family labels are shown only for groups with average abundance above 5%. The same color donates the same taxonomic group at phylum level. Different study sites are represented in different panels: a) Walagan North (WN); b) Walagan (WL); c) Tayuan (TY); d) Jiagedaqi (JQ). Following each label (WN, WL, TY and JQ) is the tag "a0, 3, 5" indicating active layer of permafrost (a), uncontaminated (0) and contaminated with 30% (v/w) (3) or 50% v/w (5) crude oil.

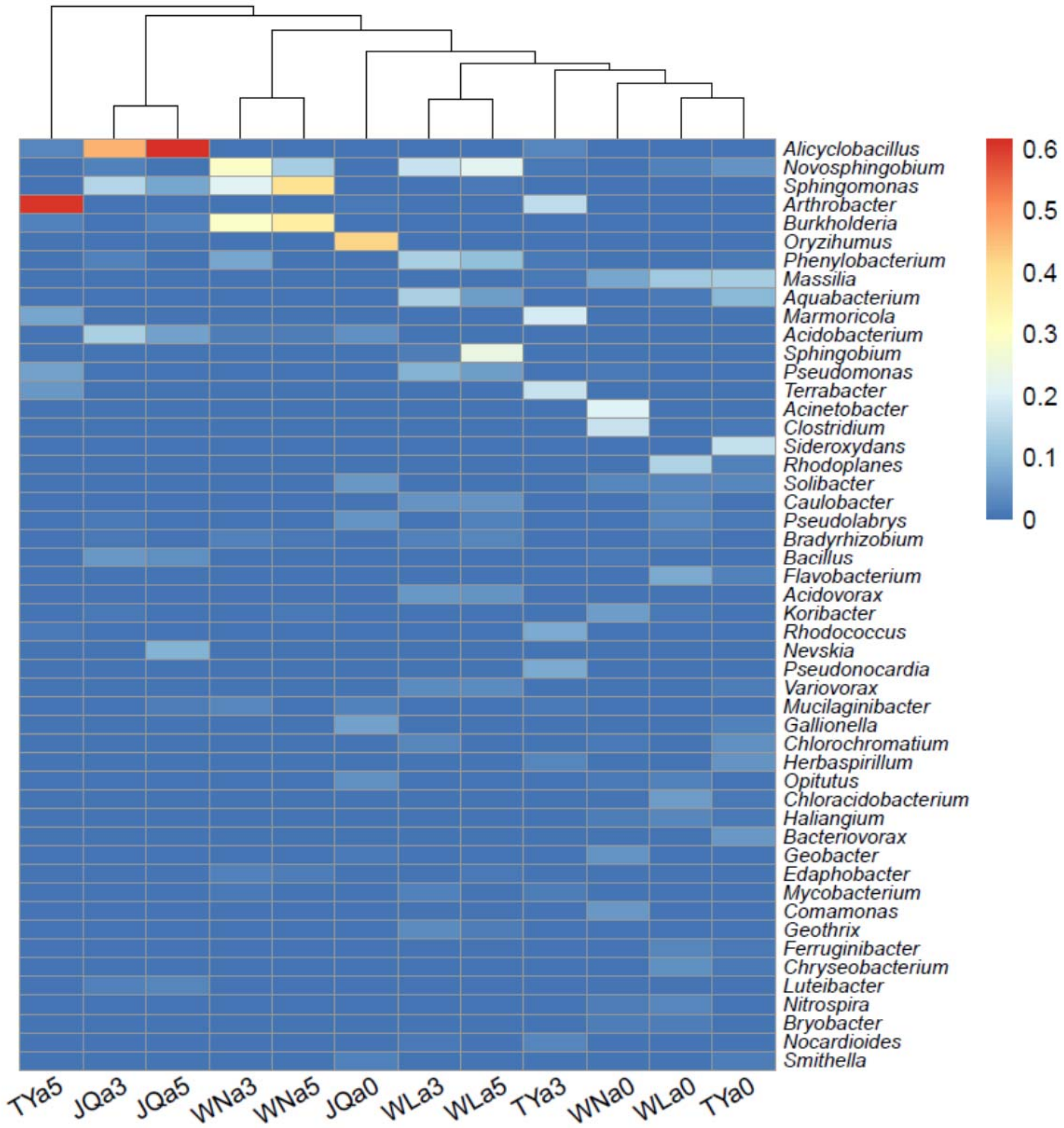

Fig. S4. Hierarchical heatmap showing the distribution of the major (top 50) bacterial groups at the genus level in samples of uncontaminated and crude oil contaminated permafrost active layer at different study sites. Study sites: a) Walagan North (WN); b) Walagan (WL); c) Tayuan (TY); d) Jiagedaqi (JQ). Following each label (WN, WL, TY and JQ) is the tag "a0, 3, 5" indicating active layer of permafrost (a), uncontaminated (0) and contaminated with 30% (v/w) (3) or 50% v/w (5) crude oil. The heatmap was constructed according to the Bray-Curtis distance and the complete clustering method. The relative values for genus abundance are indicated by colour intensity, with the legend at the top right corner.

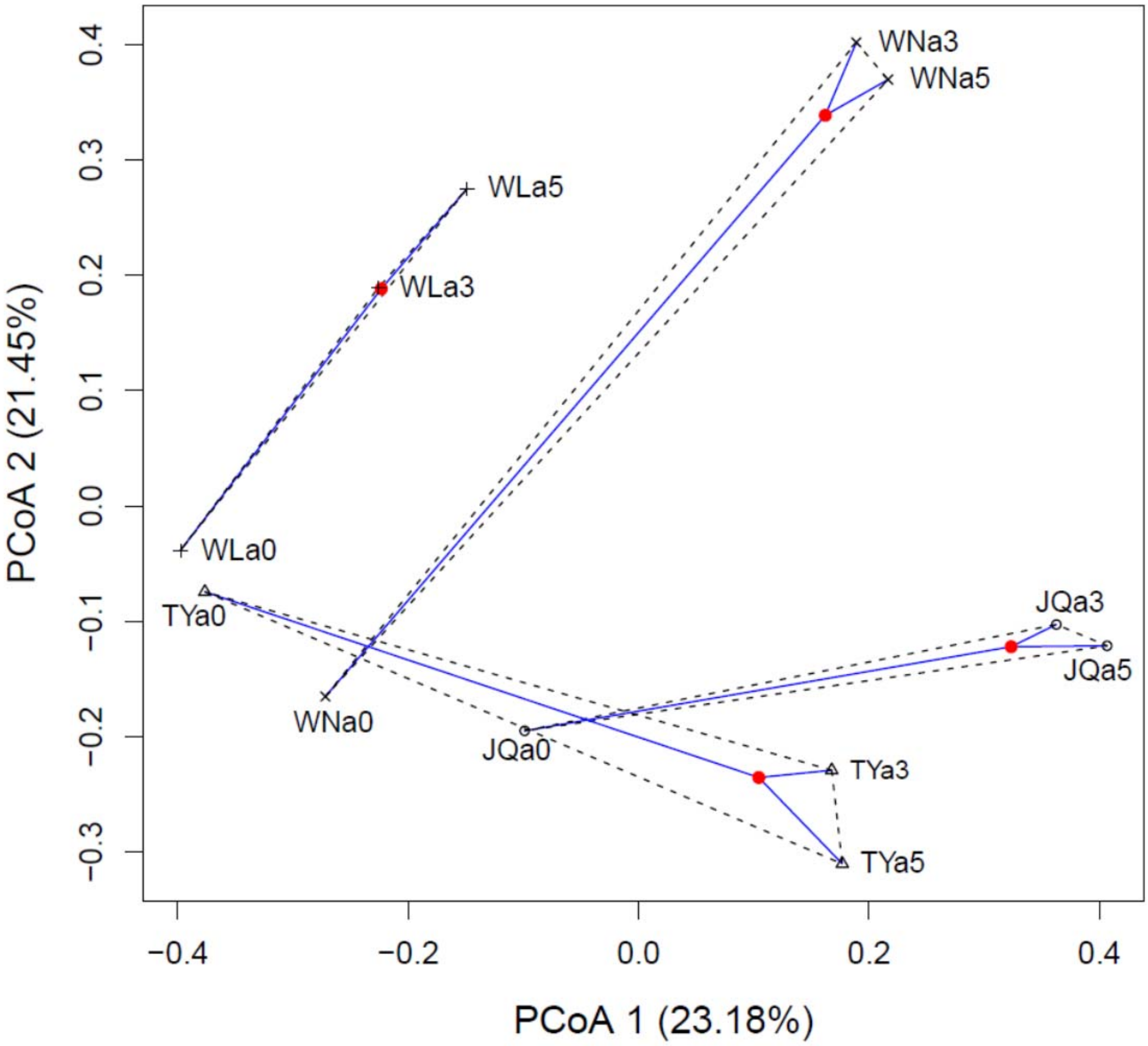

Fig. S5. Principal coordinates analysis (PCoA) showing the clustering of bacterial communities in samples of uncontaminated and crude oil contaminated permafrost active layer at different study sites. Study sites: a) Walagan North (WN); b) Walagan (WL); c) Tayuan (TY); d) Jiagedaqi (JQ). Following each label (WN, WL, TY and JQ) is the tag "a0, 3, 5" indicating active layer of permafrost (a), uncontaminated (0) and contaminated with 30% (v/w) (3) or 50% v/w (5) crude oil. The red points represent the centroid for each group in multivariate space.

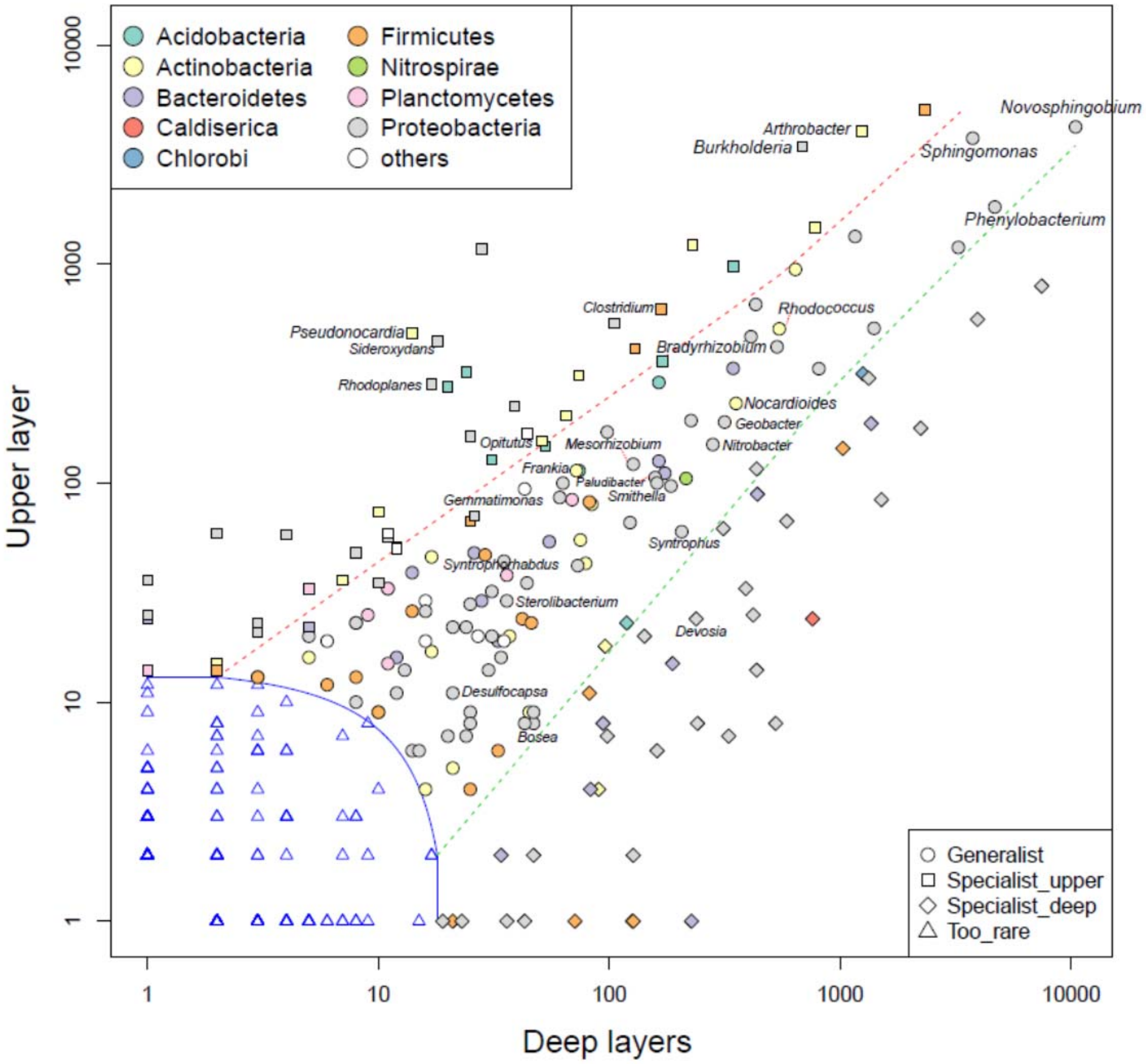

Fig. S6. Habitat generalists and specialists in relation to permafrost depth. The upper active layer samples are at 20-40 cm depth, while the deep layers donate samples from the deep active layer 70-90 cm and upper permafrost130-160 cm. The classification of generalists and specialists was done based on the function CLAM test in vegan package according to the estimated species relative abundance in samples of upper active layer, deeper active layer and upper permafrost. The test was applied with arguments of default alpha value and a specialisation threshold of 2/3 according to the supermajority rule. The x and y axes represent the abundance of different genera according to permafrost depth profile. All the counts were added by 1 to let the marginal taxa evenly arranged in the plot space. Specialists for upper active layer map at the left side of the plot, specialists for deeper layers at the right side, generalists are located in the middle and rare taxa are at the bottom left corner. Labels are only shown for representative taxa used in the habitat analysis with regard to upper/deep samples and discussed in the main text.

Table S1. Estimated OTU richness, diversity indices and sample coverage at 3% distance for the 16S rRNA gene libraries of the samples of uncontaminated and crude oil contaminated permafrost active layer. The values in parenthesis of ACE, Chao and Shannon indices show the lower and upper bound at 95% confidence intervals. The number of OTUs, alpha diversity indices and coverage were calculated on subsampled OTU data. The coverage was generated by mother platform and the other indices were summarised in R package vegan v.2.0-7.

Samples	Total OTUs	Estimated OTU richness indices			Coverage
		ACE	Chao	Shannon	
WLa0	2687	10209 (9710;10744)	6526 (6038;7085)	7.35 (7.31;7.38)	0.68
WLa3	1283	3407 (3200;3636)	2537 (2297;2835)	5.57 (5.53;5.61)	0.92
WLa5	1209	3773 (3522;4050)	2540 (2286;2854)	5.52 (5.47;5.56)	0.89
WNa0	2001	4324 (4124;4541)	3431 (3205;3698)	6.68 (6.65;6.72)	0.88
WNa3	462	1185 (1068;1324)	827 (719; 981)	3.65 (3.61;3.69)	0.97
WNa5	541	1468 (1333;1625)	1119 (957;1344)	3.8 (3.75;3.84)	0.96
TYa0	2448	8753 (8327;9211)	6102 (5600;6683)	7.05 (7.01;7.09)	0.74
TYa3	782	1548 (1442;1671)	1210 (1102;1355)	5.08 (5.04;5.12)	0.96
TYa5	636	1197 (1107;1305)	1008 (906;1150)	4.57 (4.53;4.62)	0.96
JQa0	1179	2382 (2244;2538)	2035 (1854;2264)	5.92 (5.88;5.95)	0.93
JQa3	404	938 (844;1051)	661 (578;783)	3.61 (3.57;3.65)	0.98
JQa5	433	1019 (916;1142)	797 (681;968)	4.21 (4.17;4.24)	0.97

Sample codes: WL-Walagan, WN-Walagan North, TY-Tayuan, JQ-Jiagedaqi; a-active layer of permafrost; 0-uncontaminated; 3-contaminated with 30% (v/w) crude oil; 5-contaminated with 50% (v/w) crude oil.

Table S2. Overview of potential hydrocarbon degrading genera present in the 16S rRNA gene libraries of the contaminated permafrost samples. The list is based on the ratio values calculated according to the max and min relative abundance. Only genera with ratio above 15 are shown. Max and min values correspond to the maximum and minimum nonzero relative abundance of a given genus in the gene library, and abs_min indicates the minimum observation including zero. PubMed identifiers for other scientific articles reporting on the hydrocarbon degrading capabilities of the listed genera are also included.

**Genus**	**ratio**	**max**	**min**	**abs_min**	**PMID references**
Arthrobacter	2695.01	60.64	0.023	0	4557559, 14872323, 10645629
Sphingomonas	2124.49	39.73	0.019	0	25819957, 21742816
Novosphingobium	1658.95	29.20	0.018	0.0176	19062646, 25007154, 15388699
Acinetobacter	1120.88	20.96	0.019	0	26027354, 25661008, 24070455, 24665769
Phenylobacterium	798.94	14.06	0.018	0.0176	25819957, 2658038
Terrabacter	794.19	17.87	0.023	0	25520173, 9336889
Aquabacterium	712.05	14.03	0.020	0	22684213, 23398624
Sphingobium	605.22	24.33	0.040	0	25146193, 22398031, 21862321, 25401077
Nevskia	454.9	8.96	0.020	0	10616721, 22899015
Pseudomonas	428.28	8.82	0.021	0	4557559, 12534814
Rhodococcus	205.56	7.63	0.037	0	25401077, 25747183, 25522518, 25244073
Burkholderia	199.18	36.99	0.186	0	20638971, 11770829
Pseudonocardia	197.61	7.94	0.040	0	21974888, 10446711
Flavobacterium	172.35	7.77	0.045	0	25923541, 19419068, 4557559
Variovorax	167.32	3.45	0.021	0	24430482, 21183664
Nocardioides	128.76	2.90	0.023	0	11394785, 10919761
Caulobacter	103.26	4.86	0.047	0	3571158, 11259647
Delftia	66.75	1.14	0.017	0	15618615, 24117085
Mesorhizobium	28.44	1.14	0.040	0	16085827, 21562979
Streptomyces	27.71	0.57	0.021	0	24898800, 24061563, 22425516, 21301805
Geobacter	25.24	4.74	0.188	0	12449317, 16348226
Alicyclobacillus	21.18	61.69	2.913	0	24031244
Desulfosporosinus	15.65	0.28	0.018	0	23809669, 20428224, 11211250, 12222956

Table S3. Overview of sampling sites, environmental and soil properties.

Sampling sites		Environmental properties					Soil properties				
Site name	Sample code	Site coordinates	ELV (m)	MST (°C)	MWT (°C)	MAP (mm)	pH	Water content (%)	TOC (%)	TN (%)	TP (%)
Walagan North	WNa[a]	N 52°43' E 124°30'	407	19.7	-26.2	462	5.7	42.5	27.88	1.88	0.36
Walagan	WLa[a]	N 52°26' E 124°40'	403	18.5	-26.3	462	6.3	41.1	43.89	1.27	0.12
Tayuan	TYa[a]	N 51°27 E 124°15'	581	17.6	-27.4	514	6.2	36.8	15.66	0.98	0.30
Jiagedaqi	JQa[a]	N 50°41' E 124°17'	390	19.1	-25.5	495	6.4	35.3	21.07	1.03	0.21

[a]a stands for active layer; Abbreviation: ELV, elevation; MST, mean summer temperature; MWT, mean winter temperature; MAP, mean annual precipitation; TOC, total organic carbon; TN, total nitrogen; TP, total phosphorus (all measured as gram/gram soil)
